# Absence of Autophagy-Related Proteins Expression Is Associated with Poor Prognosis in Patients with Colorectal Adenocarcinoma

**DOI:** 10.1155/2014/179586

**Published:** 2014-03-05

**Authors:** Ji Hye Choi, Young-Seok Cho, Yoon Ho Ko, Soon Uk Hong, Jin Hee Park, Myung Ah Lee

**Affiliations:** ^1^Department of Biomedical Science, The Catholic University of Korea College of Medicine, Seoul 137-701, Republic of Korea; ^2^Department of Internal Medicine, Uijeongbu St. Mary's Hospital, The Catholic University of Korea College of Medicine, Uijeongbu 480-717, Republic of Korea; ^3^Department of Pathology, Asan Medical Center, University of Ulsan College of Medicine, Seoul 138-736, Republic of Korea; ^4^Department of Internal Medicine, Seoul St. Mary's Hospital, The Catholic University of Korea College of Medicine, Seoul 137-701, Republic of Korea

## Abstract

*Background/Aim.* Autophagy, a cellular degradation process, has paradoxical roles in tumorigenesis and the progression of human cancers. The aim of this study was to investigate the expression levels of autophagy-related proteins in colorectal cancer (CRC) and to evaluate their prognostic significance. *Methods.* This study is a retrospective review of immunohistochemical and clinicopathological data. All specimens evaluated were obtained from 263 patients with colorectal cancer who had undergone surgery between November 1996 and August 2007. The primary outcomes measured were the expression levels of three autophagy-related proteins (ATG5, BECN1/Beclin 1, and Microtubule-associated protein 1 light chain 3B (LC3B)) by immunohistochemistry and its association in clinicopathological parameters and patient survival. *Results.* The autophagy-related protein expression frequencies were 65.1% (151/232) for ATG5, 71.3% (174/244) for BECN1, and 74.7% (186/249) for LC3B for the 263 patients. Correlation between the expression of autophagy-related proteins was significant for all protein pairs. Multivariate analysis showed that negative LC3B expression and absence of autophagy-related proteins expression were independently associated with poor prognosis. *Conclusion.* Absence of autophagy-related proteins expression is associated with poor clinical outcome in CRC, suggesting that these proteins have potential uses as novel prognostic markers.

## 1. Introduction

Autophagy is an evolutionarily conserved catabolic process in which the cell self-digests excessive, damaged, or aged proteins and damaged organelles [1]. This cellular process is characterized by the formation of autophagosomes, double-membraned vesicles that sequester the cytoplasmic material that is destined for degradation in the lysosome [2]. Autophagy occurs at low basal levels in most cells to maintain cellular homeostasis. However, this process is upregulated in response to metabolic stresses, including starvation, hypoxia, and growth factor deprivation, to generate intracellular nutrients and energy [1]. Autophagy is involved in the regulation of many physiological and pathological processes, including cell development and differentiation, immunity, energy homeostasis, cell death, and tumorigenesis [2]. The role of autophagy in tumorigenesis is complex and paradoxical [3]. Autophagy defects can accelerate tumorigenesis. The mammalian autophagy gene *BECN1/Beclin 1* is frequently monoallelically deleted in many cancers, including human ovarian, breast, and prostate cancers, and mice with allelic loss of *BECN1* are more likely to develop hepatocellular carcinoma (HCC), lung adenocarcinomas, lymphomas, and mammary hyperplasia [4, 5]. However, other studies have suggested that the prosurvival function of autophagy under stress conditions can promote tumor development [6–8]. In addition, the pharmacological or genetic inhibition of autophagy has been shown to enhance the cytotoxic effects of chemotherapeutic agents [9–11].

Colorectal cancer (CRC) is one of the leading causes of cancer-related mortality worldwide [12]. Although the potential of several molecular markers, including the *TP53* or *KRAS* mutational status and loss of heterozygosity in chromosome 18q, has been investigated to identify patients with a greater likelihood of recurrence after curative resection, the results are conflicting [13–15]. A previous study demonstrated that autophagy is activated in CRC *in vitro* and *in vivo*, and autophagy may contribute to the survival of colorectal cancer cells that have acquired resistance to nutrient starvation [16].

Autophagy-related genes (ATGs) regulate and implement the autophagy process, and more than 30 ATGs have been identified in yeast, of which 20 have also been found in humans [17]. Among these genes, BECN1 (a mammalian homolog of the yeast ATG6 protein) is an essential modifier of the autophagic process and has been implicated in the tumorigenesis of many types of cancers [18]. Microtubule-associated protein 1 light chain 3 (LC3) is an autophagosomal ortholog of the yeast ATG8 and exists in two forms, LC3-I and its proteolytic derivative LC3-II. The activation of autophagy in response to various stresses stimulates the conversion of LC3-I into LC3-II and upregulates LC3 expression. LC3 is a specific marker of autophagosome formation and is the most widely monitored autophagy-related protein [19]. ATG5 is a key regulatory protein involved in the early stage of autophagosome formation. The conjugation of ATG5 with ATG12 through an ubiquitin-like system contributes to autophagosome formation [20]. In the present study, we evaluated the expression of autophagy-related proteins (ATG5, BECN1, and LC3) in primary colorectal adenocarcinomas and their relationship with clinicopathological parameters and clinical outcomes.

## 2. Materials and Methods 

### 2.1. Patients and Tumor Samples

All specimens evaluated in the present study were obtained from patients with CRC between November 1996 and August 2007 at Seoul St. Mary's Hospital of the Catholic University of Korea. During the study period, a total of 298 consecutive patients with CRC who had undergone surgery were enrolled. Patients were included in the present study if the survival time was known and paraffin blocks of tumor specimens were available. Patients were excluded from this study due to surgery-related mortality (*n* = 10), poor quality of paraffin-block (*n* = 15), and loss of tissue during the construction of tissue microarray blocks (*n* = 10). Therefore, 263 pathologically confirmed specimens were investigated. None of the patients had received either radiotherapy or chemotherapy preoperatively. We reviewed the medical records of the patients and obtained clinicopathological data, including age, sex, histopathological diagnosis, and pathological tumor stage, and patient outcomes, such as last follow-up and recurrence. Cancer-related death was identified based on medical records and/or telephone interviews. The World Health Organization criteria were used for histological classification, and the TNM classification system was used for postoperative pathological staging according to the 7th edition of the American Joint Committee on Cancer staging criteria. This study was approved by the Institutional Research Ethics Board of Seoul St. Mary's Hospital of the Catholic University of Korea.

### 2.2. Tissue Microarray Methods

Tissue microarray recipient blocks were constructed from archival formalin-fixed, paraffin-embedded tissue blocks prepared from primary colorectal cancer specimens according to established methods [21]. Briefly, morphologically representative tissue areas marked on standard hematoxylin and eosin-stained sections were punched from donor blocks, and the tissue cores were placed in the recipient blocks. In total, 30 cores were assembled in a recipient block. After construction, 4 *μ*m sections were cut from the array block and transferred to glass slides.

### 2.3. Immunohistochemistry and Analysis

Using sections from the tissue microarrays, immunohistochemistry was performed using a Lab Vision Autostainer LV-1 (LabVision/Neomarkers, Fremont, CA) according to the manufacturer's protocol. The primary antibodies were rabbit polyclonal antibodies against ATG5, BECN1, and LC3B purchased from Abcam (Cambridge, UK). They were used at the following dilutions: ATG5 (1 : 2000), BECN1 (1 : 130), and LC3B (1 : 200). The samples were incubated with the primary antibodies at room temperature for 24 hours, and immunoreactivity was detected by a conventional streptavidin-biotin labeling method (LSAB2 System; Dako, Carpinteria, CA). For the negative controls, sections were treated using the same method with the exception that they were incubated with the antibody diluents instead of the primary antibodies. Immunostaining patterns were interpreted using a semiquantitative histological score according to our previous study [22]. The intensity of immunoreactivity was classified as negative (0), weak (1+), moderate (2+), or strong (3+). The percent area occupied by immunoreactive tumor cells was classified as grade 0 (0%), 1 (1%–30%), or 2 (31%–100%). A composite score was calculated by multiplying the intensity and percentage scores. In the evaluation of autophagy-related protein expression, a composite score of 0 was considered negative protein expression, and scores of 1 to 3 were defined as positive protein expression.

### 2.4. Statistical Analysis

Statistical analyses were performed using the SPSS software package (version 13.0; SPSS, Chicago, IL). The Chi-square test, Fisher's exact test, and Jonckheere-Terpstra test were used for the analysis of the relationship between the immunohistochemical profiles and the clinicopathological variables. The “time-to-event” data were evaluated by the Kaplan-Meier method, and significant differences between the groups were identified by the log-rank test. The Cox proportional hazards regression model was used to identify the factors related to overall survival (OS). All variables with a *P* < 0.2 in the univariate analysis were included in the multivariate analysis. Overall survival (OS) was calculated from the date of diagnosis to the date of death or the last follow-up. Survival rates and odds ratios are presented with their respective 95% confidence intervals (CIs). A value of *P* of <0.05 was considered statistically significant.

## 3. Results

### 3.1. Patients' Clinical Characteristics

In total, 263 paraffin blocks of tumor samples were available from patients who had undergone surgery.  Table 1 shows the clinicopathological characteristics of the patients. The patient cohort consisted of 141 males and 122 females, with a median age of 64 years (range, 30−83 years). Histologically, most patients (88.6%) had tubular adenocarcinoma. According to the American Joint Committee on Cancer staging criteria, 38 patients (14.4%) had stage I disease, 67 (25.5%) had stage II disease, 101 (38.4%) had stage III disease, and 57 (21.7%) had stage IV disease. All patients with stage IV received palliative resection for primary CRC. Tissue microarrays were constructed with samples obtained from primary CRC, not metastatic sites. One hundred and twenty (45.6%) patients underwent adjuvant chemotherapy and/or radiation therapy, 74 (28.1%) received 5-fluorouracil (FU)-based chemotherapy, and 46 (17.5%) received 5-FU-based concurrent chemoradiation or radiation alone. The median follow-up duration was 71.4 months (range, 0.5–197.4 months) after surgical resection. Among the 263 patients, 87 (33.1%) died of their tumors and 176 (66.9%) were alive at the last follow-up.

### 3.2. Expression of Autophagy-Related Proteins 

**Figure 1:**
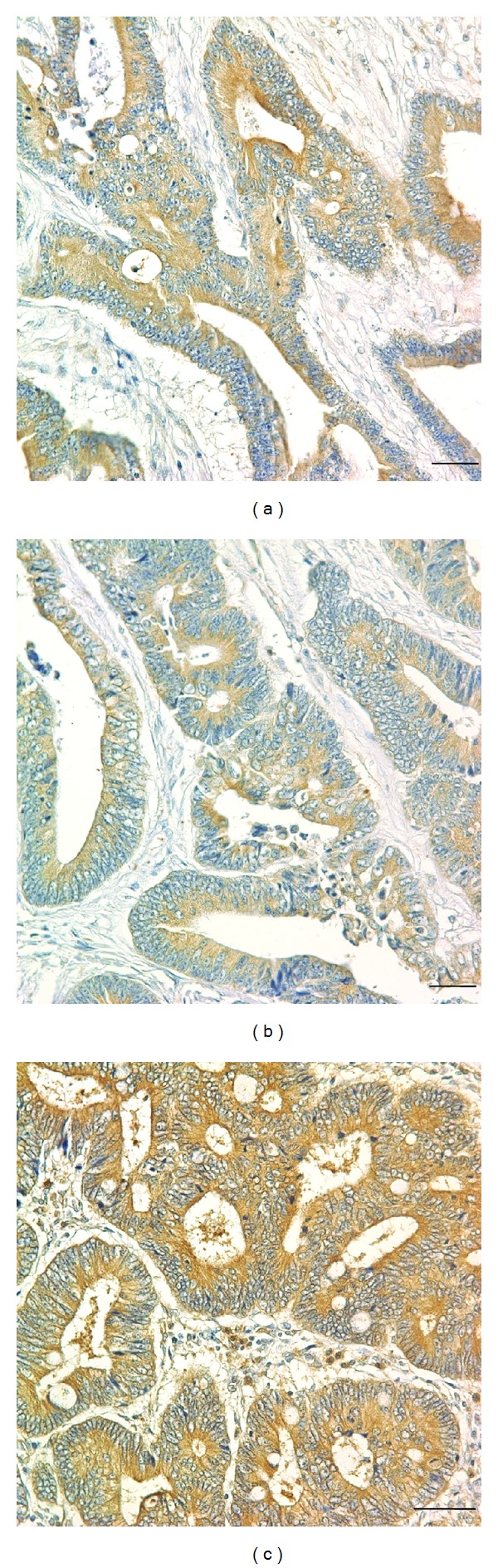
Immunohistochemistry findings show autophagy-related protein overexpression in CRC. Tumor cells show strong positive staining for ATG5 (a), BECN1 (b), and LC3B (c) proteins. Magnification, ×400. Scale bars, 50 *μ*m.

**Figure 2:**
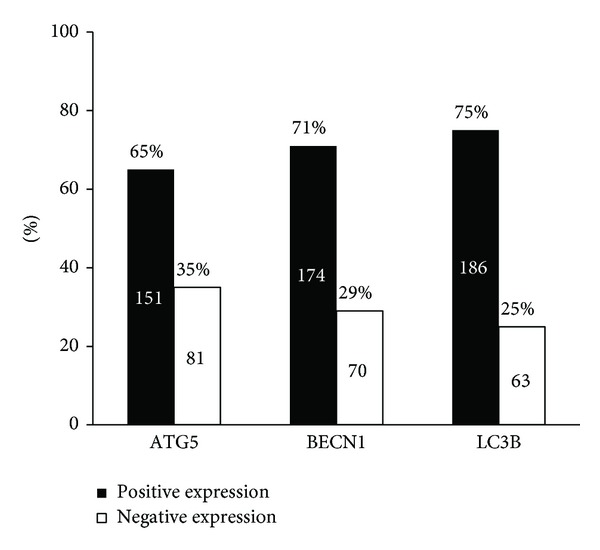
Expression levels of autophagy-related proteins were dichotomized into positive and negative categories based on the intensity and percentage of staining. Missing data for ATG5 (*n* = 31), BECN1 (*n* = 19), and LC3B (*n* = 14).

Figure 1 shows representative immunohistochemistry results. The autophagy-related protein expression frequencies were 65.1% (151/232) for ATG5, 71.3% (174/244) for BECN1, and 74.7% (186/249) for LC3B among the 263 patients (Figure 2). The expression of autophagy-related proteins in tumor cells was predominantly localized to the cytoplasm. The associations between the expression of autophagy-related proteins and clinicopathological parameters, including well-known prognostic factors such as histological differentiation, lymphovascular invasion and lymph node metastasis, were investigated (Table 2). Positive BECN1 expression was significantly correlated with good histological differentiation (*P* = 0.035). However, no significant correlation was observed between the expression levels of autophagy-related proteins and the clinicopathological parameters. In addition, the relationships between the expression patterns of different autophagy-related proteins were assessed (Table 3). The correlations between the expression patterns of autophagy-related proteins were statistically significant for all protein pairs.

### 3.3. Survival Analysis with respect to Clinicopathological Factors and Autophagy-Related Protein Expression

**Figure 3:**
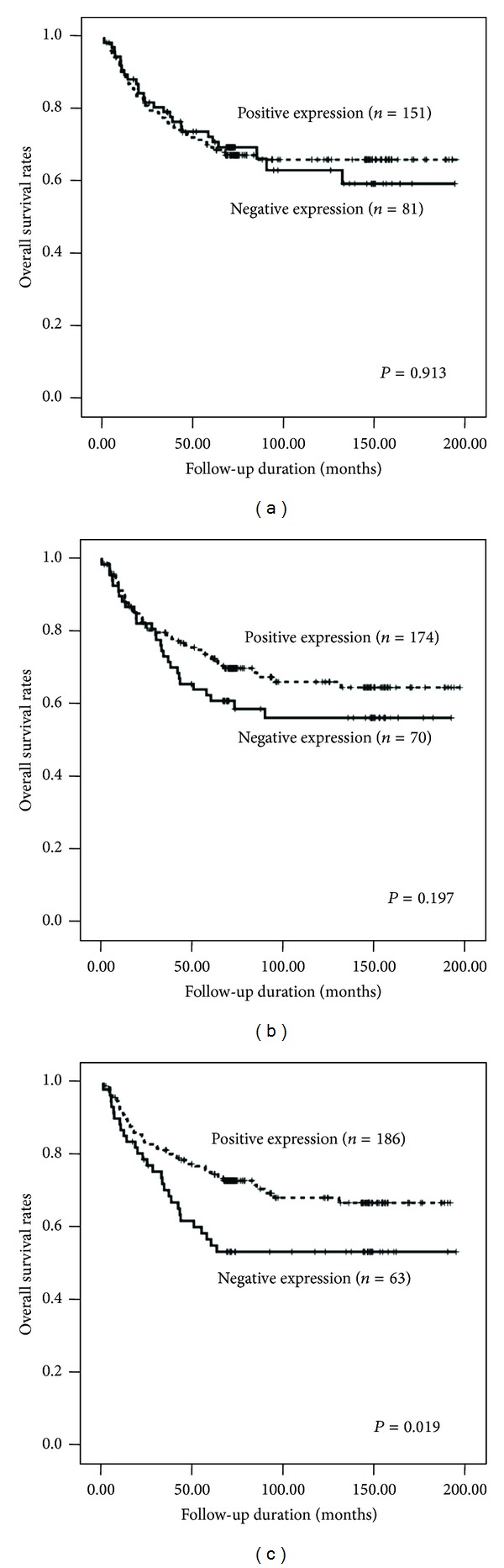
Survival curves using the Kaplan-Meier method by a log-rank test. (a) ATG5 expression. (b) BECN1 expression. (c) LC3B expression.

The overall 5-year survival rate for resected CRCs was 79.5%. The median overall survival time had not yet been reached for all patients. Univariate analysis of the clinicopathological parameters relevant to patient survival showed that the following factors were significantly associated with the overall survival of the patients: histological type (*P* = 0.004), TNM stage (*P* < 0.001), lymphovascular invasion status (*P* < 0.001), the depth of invasion (*P* < 0.001), the degree of histological differentiation (*P* = 0.001), and curative resection status (*P* < 0.001). Negative LC3B expression was found to be significantly associated with a poor outcome (*P* = 0.019; Figure 3(c)). However, the expression levels of BECN1 and ATG5 were not significantly correlated with overall survival (Figures 3(a) and 3(b)). Multivariate analysis was performed to identify relationships between the previously mentioned factors and prognosis. Curative resection status (*P* < 0.001), lymph node involvement (*P* < 0.001), and distant metastasis (*P* = 0.008) were significant poor prognostic factors. In addition, negative LC3B expression was found to be an independent indicator of poor prognosis (hazard ratio, 0.518; 95% CI, 0.319–0.841; *P* = 0.008; Table 4 and Figure 3(c)).

### 3.4. Changes in the Expression of Autophagy-Related Proteins and Their Clinical Significance

**Figure 4:**
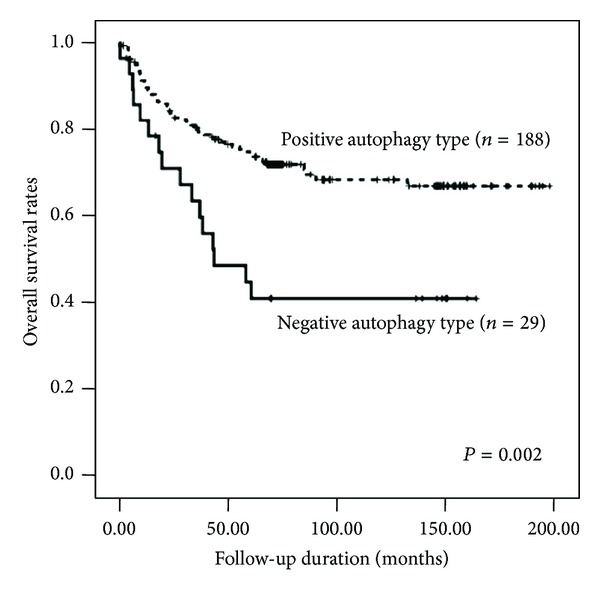
Survival curves using the Kaplan-Meier method by a log-rank test for autophagy type in all cases.

The number of autophagy-related proteins with positive expression ranged from 0 to 3 with a mean of 2.1 ± 0.7. The available 217 cases were classified into two groups according to the number of proteins that were expressed. In 29 cases, no autophagy-related protein was expressed, and these cases were defined as the negative autophagy type. The other 188 cases showed the expression of 1 to 3 proteins and were designated as the positive autophagy type. When the clinicopathological characteristics were analyzed according to these autophagy types, the negative autophagy type was found to be significantly associated with poor histological differentiation (*P* = 0.014). In the univariate analysis, patients with the negative autophagy type had a significantly worse prognosis than those with the positive autophagy type; the median overall survival time was 43.7 months (95% CI, 10.4–77.0) for negative-autophagy-type patients and had not yet been reached for positive-autophagy-type patients (*P* = 0.002). Multivariate analysis showed that curative resection status (*P* < 0.001), lymph node involvement (*P* = 0.019), and distant metastasis (*P* = 0.007) were strong predictive factors. In addition, the negative autophagy type was an independent prognostic factor (hazard ratio, 0.432; 95% CI, 0.241–0.774; *P* = 0.005; see Table 5 and Figure 4).

## 4. Discussion

Emerging evidence has shown that tumorigenesis and the progression of human cancers are affected by disturbances in the molecular machinery regulating autophagy [1, 3]. However, there are a limited number of studies of autophagy markers in CRC. The results of the present study suggest that the negative expression of various autophagy-related proteins is associated with poor clinical outcome in CRC, and the prognostic impact of these proteins seemed to be independent of well-known clinicopathological parameters. In contrast to some human cancers, including ovarian, breast, and prostate cancers, the major gastrointestinal cancers (esophageal, stomach, and colorectal cancers) exhibit high levels of autophagy activity [19, 23]. Ahn et al. reported that BECN1 protein expression, assessed by immunohistochemistry, was detected in 98 (95%) of 103 samples of colorectal adenocarcinoma and in 50 (83%) of 60 samples of gastric adenocarcinoma [23]. In this study, there was no significant association between BECN1 expression and the clinicopathological characteristics. Other previous studies have also found that autophagy-related proteins were upregulated in colon cancer, as assessed by an increase in LC3 or BECN1 expression in a significant proportion of primary tumors [16, 24–26].

The expression of autophagy-related proteins (particularly LC3 and BECN1) has been reported to be a prognostic factor in various human cancers, but the results are conflicting. Giatromanolaki et al. demonstrated that the LC3A and BECN1 proteins are highly expressed in breast, lung, endometrial, urothelial, and prostate tumors, and the expression of these proteins was significantly associated with tumor aggressiveness and poor prognosis [27]. The authors classified LC3A immunoreactivity into three types according to the staining pattern in breast carcinoma, namely, the diffuse cytoplasmic, perinuclear, and “stone-like” intracellular structure (SLS) types, each of which had a distinct prognostic relevance [28]. In colorectal adenocarcinoma, perinuclear LC3A expression, indicative of a basal level of autophagic function, has been shown to be an independent marker of good prognosis, but high SLS counts, presumably reflecting an excessive autophagic response, were associated with tumor hypoxia, metastases, and poor prognosis [29]. LC3A and B proteins are generally believed to have similar functions in the initiation and formation of autophagosomes [27]. In our study, negative LC3B expression was found to be significantly associated with poor outcomes. However, Zheng et al. reported that LC3B expression in the peripheral area of colorectal cancer tissues was significantly correlated with several clinicopathological parameters [30]. In addition, Guo et al. demonstrated that patients with low LC3 expression had a higher objective response rate among advanced colorectal cancer patients treated with cetuximab-containing chemotherapy [31]. These conflicting results could be due to the variable prognostic value of LC3, which depends on the intrinsic properties of the tumor, the stage, and the treatment regimen.

The present study showed that other key autophagy-related proteins, such as BECN1 and ATG5, were not associated with prognosis, although positive BECN1 expression was significantly correlated with good histological differentiation. BECN1 expression has been widely studied to determine its association with the prognosis of CRC. However, several studies have yielded conflicting results. Li et al. reported that high BECN1 expression was associated with favorable outcomes in resected stage IIIB colon cancers treated with 5-FU-based adjuvant chemotherapy after surgery [24]. Recently, Koukourakis et al. classified the BECN1 expression patterns in colorectal cancer patients treated with surgery alone into four categories, combining the extent and intensity of staining [25]. The four groups were the normal-like, limited overexpression, extensive overexpression, and underexpression groups. In this study, the underexpression of BECN1 was correlated with poor prognosis, whereas the extensive overexpression of BECN1 was associated with the tumor HIF-1*α* level and aggressive clinical behavior. Other studies have also linked BECN1 overexpression with reduced survival in colon cancer patients treated with adjuvant 5-FU [26] and lower BECN1 expression with a longer median progression-free survival in patients with advanced colorectal cancer treated with cetuximab-containing chemotherapy [31]. As for LC3 expression, the heterogeneity due to the mix of colon and rectal cancers as well as the variation in the stage from stage I to IV complicates the interpretation of the prognostic value of BECN1 expression in colorectal cancers [24].

Data regarding the role of autophagy-related proteins other than LC3 and BECN1 are still lacking. A recent study by Cho et al. demonstrated that ATG5 expression was found in the tumors of approximately 80% (102/124) of colorectal cancer patients, and high protein expression correlated with lymphovascular invasion, which is a risk factor for recurrence and poor survival outcome [32]. However, there was no correlation between ATG5 expression and overall survival or disease-free survival. Another study reported that ATG10, one of the E2-like conjugation enzymes for ATG12-ATG5 conjugation, was highly increased in colorectal cancer tissues, and increased protein expression was associated with lymphovascular invasion and lymph node metastasis [33].

Because the associations among the expression levels of the autophagy-related proteins (ATG5, BECN1, and LC3B) were strong and significant, consistent with their known roles in regulating autophagy activation, we divided the patients into two groups according to the number of autophagy-related protein expression changes. The positive autophagy type was associated with good histological differentiation and a favorable clinical outcome. Although the curative resection status was the strongest prognostic factor in the multivariate analysis, the change in autophagy-related protein expression was also an independent prognostic factor. Taken together, these findings suggest that the number of autophagy-related protein changes is significantly associated with tumor progression. Recently, the roles of autophagy in colorectal cancer development and treatment have been investigated both *in vitro* and *in vivo*. The transfection of the low *BECN1* gene-expressing colon cancer cells with the *BECN1* gene inhibited cell growth, and cell cycle analysis revealed G1 arrest, indicating that BECN1 plays an important role in the proliferation of colorectal cancer cells [34]. In contrast, autophagy also may play an important role in the survival of colorectal cancer cells, suggesting that the role of autophagy in colorectal cancer may be complex [16].

Sex significantly influences several clinicopathological characteristics of CRC, including incidence and mortality rates, survival chemotherapeutic response, and certain molecular characteristics [35–37]. Emerging evidence indicates that estrogens and/or progestins have a protective effect against colorectal carcinogenesis [36]. This protective effect of estrogens is mediated by interaction with estrogen receptor *β* (ER *β*), microsatellite status, and progressive hypermethylation of the CpG islands of the promoter region of the ER [37]. In the present study, there were no differences in autophagy-related proteins expression according to sex, and the distribution of conventional clinicopathological and prognostic factors did not differ between female and male CRC. However, recent *in vitro* study has demonstrated that 2-methoxyestradiol, which derives from the NADPH-dependent cytochrome P450 metabolism of 17*β*-estradiol, is able to induce apoptosis as well as autophagy in colon carcinoma-derived cell lines [38]. This result suggests that autophagy could play a role for gender differences in CRC. Further research for the exact molecular mechanisms is necessary.

Studies regarding autophagy in cancer tissue sections are limited by the difficulty in performing dynamic assays, which are necessary for monitoring autophagy. Although counting autophagic vesicles by electron microscopy is the standard approach for detecting autophagy, this method is not readily accessible and is not capable of evaluating entire tumor samples [39]. Immunohistochemical staining is a useful method for monitoring autophagic activity in surgically resected cancer specimens, and the advantages of this method include the ability to analyze changes in the expression levels of a number of autophagy-related proteins. The p62/sequestosome 1 (SQSTM1), a cytosolic adaptor protein, facilitates the autophagic degradation of ubiquitinated protein aggregates in lysosomes [40]. The impaired turnover of p62 due to defective or impaired autophagy is associated with the accumulation of p62 [41]. p62 can also be used as a protein marker of autophagy [39]. In a future study, adding p62 would be helpful to improve prognostic role of autophagy-related proteins. This study is also limited by the small number of patients, and the results cannot be considered representative of all colorectal cancer patients. To determine the role of changes in autophagy-related protein expression levels as prognostic factors, further validation and standardization of the immunohistochemical assay are required, in addition to a larger number of colorectal cancer patients.

In conclusion, we found that negative LC3B expression and absence of autophagy-related proteins expression were independently associated with poor survival in patients with CRC. Because the expression levels of autophagy-related proteins showed no correlation with well-known prognostic markers, these proteins have potential uses as novel prognostic markers in patients with colorectal cancer.

## Figures and Tables

**Table 1:** Baseline clinicopathological characteristics of patients with colorectal cancer.

Characteristics	Total	
	Patient number	%
Patient number	263	
Age (years), median (range)	64 (30–83)	
<65	141	53.6
≥65	122	46.4
Gender		
Male	141	53.6
Female	122	46.4
Histology		
Tubular adenocarcinoma	233	88.6
Others*	30	11.4
T stage		
1	1	0.4
2	51	19.4
3	163	62.0
4	48	18.2
N stage		
0	114	43.3
1	80	30.4
2	69	26.2
Stage		
I	38	14.4
II	67	25.5
III	101	38.4
IV	57	21.7
Histological grade		
Well/moderately	222	84.4
Poorly	41	15.6
Lymphovascular invasion		
Negative	99	37.6
Positive	164	62.4
Site of primary tumor		
Right colon	69	26.2
Left colon	83	31.6
Rectum	111	42.2
Curative resection (R0 resection)		
Yes	208	79.1
No	55	20.9
Adjuvant therapy (*n* = 120)		
5-FU-based chemotherapy	74	28.1
5-FU-based CCRT	46	17.5

*Mucinous carcinoma (*n* = 28), signet-ring-cell carcinoma (*n* = 2).

Data are numbers of patients with percentages in parentheses unless otherwise specified.

SD: standard deviation; T1: tumor invades submucosa; T2: tumor invades muscularis propria; T3: tumor invades through the muscularis propria into the subserosa or into the nonperitonealized pericolic or perirectal tissues; T4: tumor perforates the visceral peritoneum or directly invades other organs; N1: metastasis in 1–3 pericolic or perirectal lymph nodes; N2: metastasis in >3 pericolic or perirectal lymph nodes; 5-FU: 5-fluorouracil, CCRT: concurrent chemoradiation therapy.

**Table 2:** Relationships among clinicopathological factors and the expression patterns of autophagy-related proteins.

	ATG5		BECN1		LC3B	
	Negative, *n* (%)	Positive, *n* (%)	Negative, *n* (%)	Positive, *n* (%)	Negative, *n* (%)	Positive, *n* (%)
T stage						
1-2	20 (39.2)	31 (66.7)	14 (28.0)	36 (72.0)	8 (16.3)	41 (83.7)
3	46 (33.8)	90 (66.2)	46 (30.9)	103 (69.1)	43 (27.9)	111 (72.1)
4	15 (33.3)	30 (33.7)	10 (22.2)	35 (77.8)	12 (26.1)	34 (73.9)
*P*		0.578		0.444		0.152
N stage						
0	38 (38.0)	62 (62.0)	35 (34.0)	68 (66.0)	28 (25.9)	80 (74.1)
1	25 (35.2)	46 (64.8)	23 (29.9)	54 (70.1)	21 (27.6)	55 (72.4)
2	18 (29.5)	43 (70.5)	12 (18.8)	52 (81.3)	14 (21.5)	51 (78.5)
*P*		0.290		0.044		0.695
Distant metastasis						
No	68 (38.2)	110 (61.8)	57 (30.0)	133 (70.0)	49 (25.0)	147 (75.0)
Yes	13 (24.1)	41 (75.9)	13 (24.1)	41 (75.9)	14 (26.4)	39 (73.6)
*P*		0.056		0.396		0.833
Stage						
I	14 (37.8)	23 (62.2)	11 (30.6)	25 (69.4)	6 (16.7)	30 (83.3)
II	22 (40.7)	32 (59.3)	23 (39.0)	36 (61.0)	20 (31.3)	44 (68.8)
III	32 (36.8)	55 (63.2)	23 (24.2)	72 (75.8)	23 (24.0)	73 (76.0)
IV	13 (24.1)	41 (75.9)	13 (24.1)	41 (75.9)	14 (26.4)	39 (73.6)
*P*		0.104		0.122		0.761
Histology						
Tubular	72 (35.0)	134 (63.0)	60 (27.8)	156 (72.2)	55 (25.0)	165 (75.0)
Others^†^	9 (34.6)	17 (65.4)	10 (35.7)	18 (64.3)	8 (27.6)	21 (72.4)
*P*		0.976		0.382		0.763
Lymphovascular invasion						
Negative	33 (37.9)	54 (62.1)	30 (33.7)	59 (66.3)	24 (26.1)	68 (73.9)
Positive	48 (33.1)	97 (66.9)	40 (25.8)	115 (74.2)	39 (24.8)	118 (75.2)
*P*		0.455		0.189		0.827
Histological grade						
Well/moderately	66 (33.5)	131 (66.5)	53 (26.0)	151 (74.0)	49 (23.4)	160 (76.6)
Poorly	15 (42.9)	20 (57.1)	17 (42.5)	23 (57.5)	14 (35.0)	26 (65.0)
*P*		0.285		0.035		0.124

*Statistically significant (*P* < 0.05).

^†^Mucinous carcinoma (*n* = 28), signet-ring-cell carcinoma (*n* = 2).

**Table 3:** Relationships among the expression patterns of autophagy-related proteins.

	LC3B		BECN1	
	Negative (%)	Positive (%)	Negative (%)	Positive (%)
ATG5				
Negative (%)	37 (47.4)	41 (52.6)	43 (53.8)	37 (46.3)
Positive (%)	17 (11.6)	130 (88.4)	15 (10.5)	128 (89.5)
*P*		<0.001		<0.001
Beclin-1				
Negative (%)	37 (56.9)	28 (43.1)		
Positive (%)	20 (12.0)	147 (88.0)		
*P*		<0.001		

**Table 4:** Multivariate analysis of the clinicopathological parameters and three autophagy-related proteins by overall survival rate using the Cox proportional hazards model.

Characteristics	Hazard ratio	95% CI	*P*
LC3B expression (positive versus negative)	0.518	0.319–0.841	0.008
Lymph node involvement (yes versus no)	4.349	2.275–8.313	<0.001
Curative resection (no versus yes)	4.322	2.177–8.582	<0.001
Distant metastasis (yes versus no)	2.504	1.273–4.924	0.008

Data calculated using the Cox proportional hazards model. CI: confidence interval.

**Table 5:** Predictive factors of survival by multivariate analysis using autophagy score (Cox proportional hazards model).

Characteristics	Hazard ratio	95% CI	*P*
Autophagy score (1–3 versus 0)	0.432	0.241–0.774	0.005
Lymph node involvement (yes versus no)	2.734	1.184–6.316	0.019
Curative resection (no versus yes)	3.846	1.950–7.584	<0.001
Distant metastasis (yes versus no)	2.528	1.282–4.987	0.007

Data calculated using the Cox proportional hazards model. CI: confidence interval.
